# Knowledge, Attitudes, and Perceptions of Women of Reproductive Age Regarding Fertility and Elective Oocyte Cryopreservation: A Study from the Al-Qassim Region

**DOI:** 10.7759/cureus.69903

**Published:** 2024-09-22

**Authors:** Zaheera Saadia, Reema Mohammed Alharbi, Najd Khalaf Alanazi, Ghaida Saleh Alabdulaaly, Majd Sulaiman Alsaqabi, Wojoud Abdullah Alharbi, Reem Mohammed Albarrak, Yaqeen Fahad Alrubaish

**Affiliations:** 1 Obstetrics and Gynaecology, Qassim University, Buraydah, SAU; 2 College of Medicine, Qassim University, Buraydah, SAU; 3 Obstetrics and Gynaecolgy, Qassim University, Buraydah, SAU

**Keywords:** cryopreservation, egg freezing, fertility preservation, health literacy, infertility, reproductive outcome

## Abstract

Background: Oocyte cryopreservation is used for fertility preservation, medical reasons, and social reasons to overcome physiological decline in fertility with age. There was no research to find the knowledge, attitude, and perception of women regarding oocyte cryopreservation in our region. This study thus aimed to investigate the knowledge, attitudes, and perceptions of women of reproductive age in Al-Qassim, Saudi Arabia, regarding fertility and elective oocyte cryopreservation.

Methods: An Arabic-language online survey was conducted over six months targeting women aged 18-45 years in the Al-Qassim region. A total of 612 participants completed the questionnaire, which covered topics such as reproductive aspirations, knowledge of fertility, knowledge of elective oocyte cryopreservation, and concerns regarding fertility preservation. Data analysis was performed using IBM SPSS Statistics for Windows, Version 21.0 (Released 2012; IBM Corp., Armonk, New York, United States).

Results: The majority of participants (47.1%) were aged 18-25 years. Participants' perceptions towards fertility preservation and social egg freezing were mixed, indicating varying levels of awareness about fertility issues. A statistically significant relationship was found between education level and knowledge of oocyte cryopreservation (p-value=0.004). Women with higher education levels (bachelor’s, master’s, and PhD degrees) demonstrated significantly greater knowledge (p-value=0.004) about oocyte cryopreservation compared to those with lower education levels. The study highlights the influence of educational attainment on knowledge of oocyte cryopreservation.

Conclusion: These findings align with broader trends in health education, suggesting that higher educational attainment correlates with better fertility health literacy. Enhancing fertility health literacy through educational programs may lead to more proactive health behaviours and better awareness about available assisted reproductive technologies.

## Introduction

Oocyte cryopreservation (OC) is a way to preserve gametes by freezing them for future use and preserving fertility [1]. OC started as a method to preserve the oocyte for women with medical or surgical diseases that decreased their fertility. For example, it helped women with cancer to freeze their oocytes before starting their treatment and for women undergoing surgeries that would render them infertile [2]. However, the concept of cryopreservation did not stop at only helping women with medical-related reasons but also helped to overcome physiological decline in fertility with age which is also known as ‘social reason’ or ‘non-medical reason’ [3,4].

OC was started in 1986 as an experimental method by the American Society for Reproductive Medicine (ASRM) but in 2012 the scope was extended for patients who underwent gonadotoxic therapy, and in 2014 a sheet was published on the patient education website of ASRM about the ability to use OC for women without the need for fertility-threatening disease to be an indication for OC [5]. Besides this, the use of OC extended from its use in infertility to its use in fertile women as an elective method of cryopreservation [6].

Infertility is a major issue for couples around the world with 8-12% of couples in their reproductive age suffering from infertility and in some regions, it could go up to 30% [7]. In a United Kingdom study assessing the awareness of women of reproductive age regarding fertility and elective OC for age-related fertility decline, 5482 participants were involved. The findings indicate that 73.2% were aware of OC, and 65.8% expressed willingness to undergo the procedure for age-related fertility concerns. Notably, the age group most open to the procedure was 31-35 years (45.9%) [8]. A study from the United States focused on 278 young graduates about their awareness regarding fertility preservation, OC, and reproductive resource choices. The results indicated that 64% had an average knowledge of fertility, with 87.1% acquiring this knowledge through formal education. About 93.9% of participants were aware of OC, with 63.4% obtaining information from the media. Interestingly, only 7.2% of these females had considered using OC [9]. In an Italian study involving 930 female students from the University of Padova, the investigation aimed to understand their awareness and potential intentions regarding OC. Findings reveal that 34.3% were aware of it and knew about its non-medical applications, with only 19.5% considering its use [10].

To the best of our knowledge, there isn’t any research done to study the knowledge, attitude, and perception (KAP) of women of reproductive age toward OC in the Qassim region. So, our study aims to contribute valuable insights to the understanding of how knowledgeable women in this region are about fertility preservation options and their perceptions regarding such options. The findings shed light on awareness levels, attitudes toward the procedure, and potential factors influencing decision-making. This will help us in improving awareness about assisted reproductive technology (ART) in the general population.

## Materials and methods

A cross-section study was conducted on a sample of females of reproductive age in the Qassim region via an online questionnaire. An Arabic version of the online survey was conducted through social media websites including WhatsApp (Meta Platforms, Inc., Menlo Park, California, United States) and Telegram messenger (Telegram Group Inc., Dubai, United Arab Emirates). The duration of the study was from March 15, 2024, to May 15, 2024. The study was approved by the IRB of the Ministry of Health Qassim region (approval number: 607/45/14880). All study participants were informed of the study objectives and confidentiality before consenting to participate in the survey. Participants were informed that their identities will remain confidential, and the results will only be used for research purposes. The Helsinki Declaration's guideline for research involving human subjects was followed during the study's execution.

The inclusion criteria included women from the general population aged 18-50 years from the Qassim region and any women under 18 or above 50 and any women not from Qassim regions were excluded.

An online questionnaire was developed using the Google Forms platform (Google LLC, Mountain View, California, United States) to reach many participants quickly and cost-effectively. Social media platforms WhatsApp and Telegram Messenger were used to distribute the Google Forms link. Initially, the questionnaire was administered in Arabic, then it was translated back to English. The questionnaire was developed by Kasaven et al. in 2023 [8] and we received permission to reuse the questionnaire via email. There were six categories included in the survey. Respondents first provided answers to questions about their age, ethnicity, educational background, work status, and marital status, among other sociodemographic details. Questions evaluating present reproductive intentions, including plans for future children, came next. The respondent's knowledge of fertility was evaluated in the third component, and their perceptions of fertility were evaluated in the fourth. Regarding OC for age-related fertility decline, attitudes and perceptions were evaluated in the fifth segment, while knowledge of the process was evaluated in the sixth. The data collection tool/questionnaire is given in the Appendices.

The sample size was calculated by ss =(Z2×p×q)/d2. Where ss = sample size, Z= 1.96, p= 0.5, q=(1-p) =0.5, and d= sampling error at 5%. According to this equation, we found that the lowest acceptable sample size to achieve a study with ±4% error and 95% confidence interval (CI) was 384. However, we added a safety margin and increased the sample size to at least 612.

The data was cleansed, reviewed for completeness, and checked for discrepancies to remove any potential inaccuracies. The data was analysed using IBM SPSS Statistics for Windows, Version 21.0 (Released 2012; IBM Corp., Armonk, New York, United States). The data was first coded and then entered into the software program. Following that, the proper descriptive statistics were run, and the results were distilled into frequency, percentage, and mean.

## Results

Most of the participants (n=288; 47.1%) were in the age group of 18-25 years. In addition, most of the participants (n=462; 75.5%) held bachelor's degrees, whereas only a minor percentage (n=3; 0.5%) had no formal education. Regarding employment, most of the participants (n=210; 34.3%) were either employed or students (n=236; 38.6%), with 156 (25.5%) being unemployed. The majority of participants (n=345; 56.4%) were single, while 257 (42.0%) were married. Very few individuals were widowed (n=8; 1.3%) and divorced (n=2; 0.3%), respectively, and 100% of the participants (n=612) were residents of Qassim (Table 1).

**Table 1:** Socialdemographical information of the participants (N=612)

Variables	Category	Frequency (Percentage)
Age	18-25 years	288 (47.1%)
	26-30 years	70 (11.4%)
	31-35 years	68 (11.1%)
	36-39 years	55 (9.0%)
	40-45 years	80 (13.1%)
	46-50 years	51 (8.3%)
Education	No formal education	3 (0.5%)
	Secondary school and below	78 (12.7%)
	Diploma	32 (5.3%)
	Bachelor’s degree	462 (75.5%)
	Master’s degree	29 (4.7%)
	PhD	8 (1.3%)
Employment	Unemployed	156 (25.5%)
	Employed	210 (34.3%)
	Student	236 (38.6%)
	Retired	10 (1.6%)
Marital Status	Single	345 (56.4%)
	Married	257 (42.0%)
	Divorced	8 (1.3%)
	Widowed	2 (0.3%)
Place of Residence	Qassim	612 (100.0%)
	Non-Qassim	0 (0.0%)

A total of 243 (39.7%) participants reported having children. The mean number of children was 4.45, with a standard deviation of 1.35. Additionally, 417 participants (68.1%) indicated that they would like to have children. Of those surveyed, 192 (31.3%) expressed their desire to conceive their first child between the ages of 25-29 years. On the other hand, 209 (34.2%) indicated that they would like to have their last child at 36-39 years. Moreover, 57 (9.3%) participants revealed that they had been diagnosed with a medical condition that could interfere with their ability to conceive (Table 2).

**Table 2:** Participant’s reproductive history of participants (N=612) Data has been presented as n (%), except for number of children, which has been presented as mean±SD.

Variables	Category	n (%)
Do you have children?	Yes	243 (39.7%)
	No	369 (60.3%)
How many children do you have?	Mean±SD	4.45±1.35
Do you wish to have children in future?	Yes	417 (68.1%)
	No	105 (17.2%)
	Unsure	90 (14.7%)
What is your desired age to have your first child (if not yet had children)?	20-24	26 (4.3%)
	25-29	192 (31.3%)
	30-35	93 (15.2%)
	36-39	14 (2.3%)
	40-45	0 (0%)
	>45	1 (0.2%)
	Unsure	43 (7 %)
	I had my 1^st^ child	243 (39.7%)
What is your desired age to have your last child?	20-24	4 (0.7%)
	25-29	12 (2.0%)
	30-35	104 (17.0%)
	36-39	209 (34.2%)
	40-45	126 (20.6%)
	>45	7 (1.1%)
	Unsure	150 (24.5%)
Have you ever been diagnosed with a health problem that could compromise your fertility?	Yes	57 (9.3%)
	No	501 (81.9%)
	Unsure	54 (8.8%)

A total of 42 respondents (6.9%) thought women were most fertile at 15-19 years, 218 (35.6%) thought it was at 20-24 years, and 182 (29.7%) felt it was at 25-29 years. None selected 40-45 years. Further, 330 (53.9%) participants answered that the average age at which a woman's fertility starts to fall was 40-45 years, while 176 (28.8%) answered that it was at 36-39 years. After a year of unprotected sexual activity, 213 (34.8%) respondents felt the likelihood of a 30-year-old woman getting pregnant was 80-100%, while only 13 (2.1%) said it was less than 10%. A total of 51 (8.3%) thought the chances were between 10-19% for a woman in her 40s, whereas 136 (22.2%) thought the odds were between 20-39%. Of the participants, 486 (79.4%) correctly indicated that a doctor would designate a pregnant woman as "high risk" due to her advanced maternal age if her age was 40-45 years. Moreover, 536 (87.6%) knew that women who conceived later in life were more likely to experience high blood pressure during pregnancy, blood clots in the legs and lungs, and premature birth (Table 3).

**Table 3:** Participant’s knowledge of fertility (N=612)

Variables	Category	Frequency (Percentage)
What age are women most fertile?	15-19 years	42 (6.9%)
	20-24 years	218 (35.6%)
	25-29 years	182 (29.7%)
	30-35 years	77 (12.6%)
	36-39 years	3 (0.5%)
	40-45 years	0 (0.0%)
	Unsure	90 (14.7%)
At what age on average do you think a woman’s fertility begins to decline?	15-19 years	3 (0.5%)
	20-24 years	7 (1.1%)
	25-29 years	6 (1.0%)
	30-35 years	50 (8.2%)
	36-39 years	176 (28.8%)
	40-45 years	330 (53.9%)
	Unsure	40 (6.5%)
What are the chances a woman of 30 years old will become pregnant after one year of unprotected sexual intercourse?	<10%	13 (2.1%)
	10-19%	14 (2.3%)
	20-39%	51 (8.3%)
	40-59%	42 (6.9%)
	60-79%	131 (21.4%)
	80-100%	213 (34.8%)
What are the chances a woman of 40 years old will become pregnant after a year of unprotected sexual intercourse?	<10%	29 (4.7%)
	10-19%	51 (8.3%)
	20-39%	136 (22.2%)
	40-59%	80 (13.1%)
	60-79%	88 (14.4%)
	80-100%	38 (6.2%)
From what age do you think doctors consider a pregnant woman ‘high risk’ due to advanced maternal age?	15-19 years	22 (3.6%)
	20-24 years	4 (0.7%)
	25-29 years	4 (0.7%)
	30-35 years	9 (1.5%)
	36-39 years	21 (3.4%)
	40-45 years	486 (79.4%)
	Unsure	66 (10.8%)
Are you aware that getting pregnant at an advanced maternal age can be associated with greater risk of high blood pressure in pregnancy, blood clots in legs and lungs, and your baby being born prematurely?	Yes	536 (87.6%)
	No	23 (3.8%)
	Unsure	53 (8.7%)

A total of 536 (87.6%) participants agreed that an underlying medical condition can severely reduce a woman's reproductive years. Just 135 (22.1%) of the participants indicated concern about their falling fertility as a result of ageing or a health issue. When asked how important it was to have their own biological child, 459 (75.0%) participants gave a strong response. Despite the fact that there are greater risks associated with becoming pregnant later in life, only 104 (17.0%) respondents stated they are not prepared to become pregnant later in life. Moreover, 320 (52.3%) participants said that gynaecologists and general practitioners should initiate family planning discussions with female patients who are of reproductive age, even if the patient chooses not to bring it up. Regarding the degree of awareness regarding options for fertility preservation, less than half of the participants (n=173; 28.2%) felt knowledgeable (Table 4).

**Table 4:** Perceptions of fertility preservation (N=612)

Variables	Category	Frequency (Percentage)
Can a woman’s number of reproductive years be significantly shortened by an underlying health condition?	Yes	536 (87.6%)
	No	23 (3.8%)
	Unsure	53 (8.7%)
I am currently worried about my declining fertility due to advanced age or a health-related problem:	Strongly agree	41 (6.7%)
	Agree	94 (15.4%)
	Neither agree nor disagree	209 (34.2%)
	Disagree	140 (22.9%)
	Strongly disagree	128 (20.9%)
Having my own biological child is important to me:	Strongly agree	259 (42.3%)
	Agree	200 (32.7%)
	Neither agree nor disagree	105 (17.2%)
	Disagree	27 (4.4%)
	Strongly disagree	21 (3.4%)
I am prepared to become pregnant at a later age, despite the additional risks associated with advanced maternal age during pregnancy:	Strongly agree	30 (4.9%)
	Agree	74 (12.1%)
	Neither agree nor disagree	153 (25.0%)
	Disagree	203 (33.2%)
	Strongly disagree	152 (24.8%)
Gynaecologists/General practitioners should initiate discussion regarding family planning with all female patients of reproductive age, even if the patient does not mention it herself:	Strongly agree	128 (20.9%)
	Agree	192 (31.4%)
	Neither agree nor disagree	200 (32.7%)
	Disagree	57 (9.3%)
	Strongly disagree	20 (3.3%)
I currently feel well-informed regarding the options available to me to preserve my fertility in case of age or health-related fertility decline:	Strongly agree	37 (6.0%)
	Agree	136 (22.2%)
	Neither agree nor disagree	247 (40.4%)
	Disagree	144 (23.5%)
	Strongly disagree	48 (7.8%)

**Figure 1:**
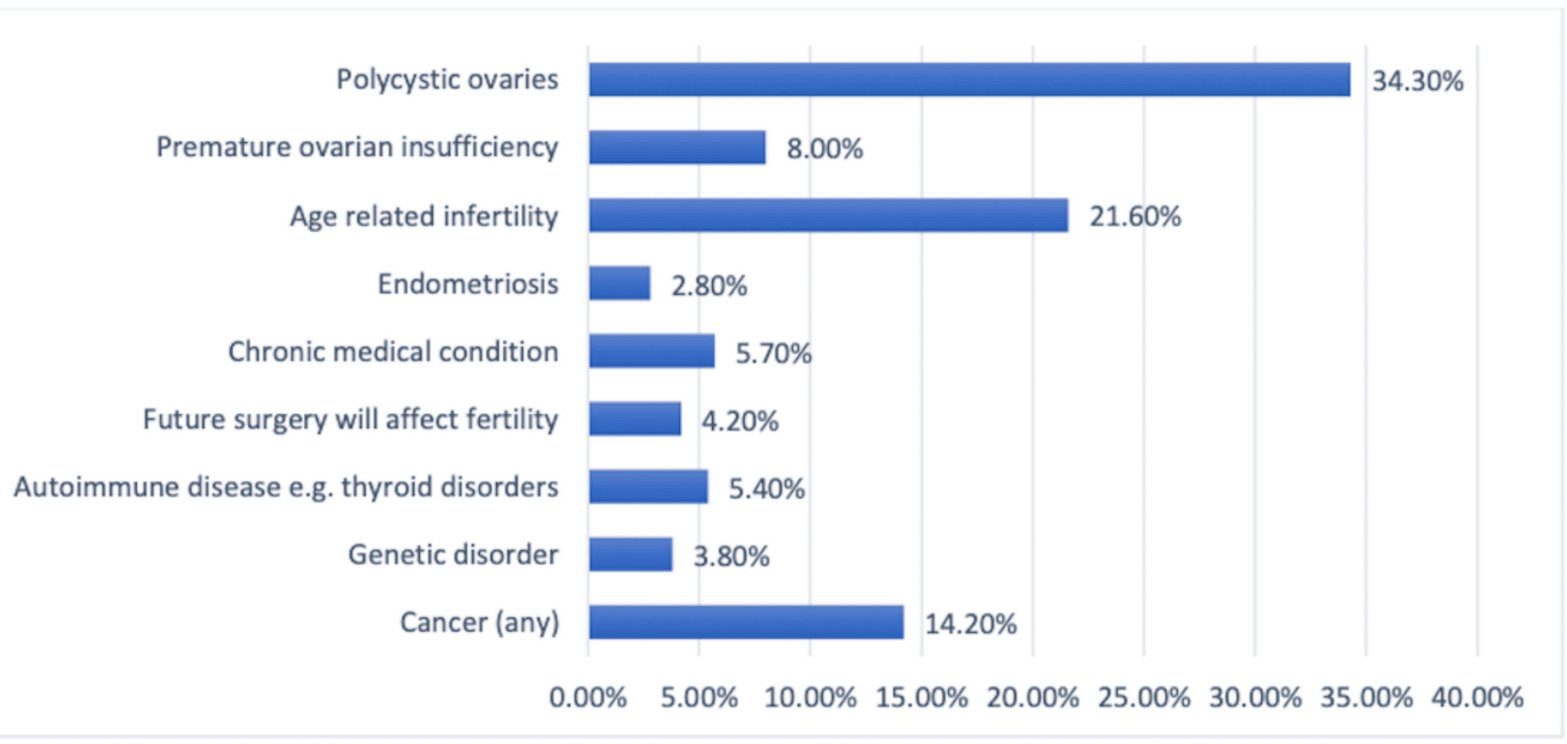
Perceived reasons for seeking fertility preservation in Saudi Arabia.

A total of 210 (34.3%) women want fertility preservation mostly because they have polycystic ovaries. Age-related infertility comes in second with 132 (21.6%) cases. Additional causes include cancer (any kind) (n=87; 14.2%), premature ovarian insufficiency (n=49; 8.0%), chronic illness (n=35; 5.7%), autoimmune disease, such as thyroid diseases (n=33; 5.4%), impending surgery that will impair fertility (n=26; 4.2%), genetic disorder (n=23; 3.8%), and endometriosis (n=17; 2.8%) (Figure 1).

Of the respondents, 539 (88.1%) had heard about social egg freezing. Regarding the idea of storing eggs to stop age-related declines in fertility, 144 (23.5%) agreed and 74 (12.1%) strongly agreed. In reference to the possibility that social egg freezing might lessen the pressure to procreate, 180 (29.4%) agreed and 93 (15.2%) highly agreed. When asked if the Ministry of Health should allow social egg freezing for everyone, 352 (57.5%) responded in the affirmative. Regarding the idea of using social egg freezing to postpone having children, 74 (12.1%) agreed and 23 (3.8%) highly agreed. Regarding social egg freezing in order to concentrate on their job, 39 (6.4%) strongly agreed and 80 (13.1%) agreed. When asked whether they would think about social egg freezing so they might have the chance for financial security before becoming mothers, 41 (6.7%) strongly agreed and 104 (17.0%) agreed (Table 5).

**Table 5:** Knowledge of elective oocyte cryopreservation (N=612)

Variables	Category	Frequency (Percentage)
Have you heard of social egg freezing?	Yes	539 (88.1%)
	No	73 (11.9%)
I would consider freezing my eggs to prevent age-related fertility decline.	Strongly agree	74 (12.1%)
	Agree	144 (23.5%)
	Neither agree nor disagree	162 (26.5%)
	Disagree	165 (27.0%)
	Strongly disagree	67 (10.9%)
If social egg freezing was available to me, it would reduce the pressure for me to have children.	Strongly agree	93 (15.2%)
	Agree	180 (29.4%)
	Neither agree nor disagree	207 (33.8%)
	Disagree	88 (14.4%)
	Strongly disagree	44 (7.2%)
Should social egg freezing be made accessible to all by the Ministry of Health?	Yes	352 (57.5%)
	No	103 (16.8%)
	Unsure	157 (25.7%)
Would you consider social egg freezing if you want to delay having a child?	Strongly agree	23 (3.8%)
	Agree	74 (12.1%)
	Neither agree nor disagree	143 (23.4%)
	Disagree	252 (41.2%)
	Strongly disagree	120 (19.6%)
Would you consider social egg freezing to focus on your career?	Strongly agree	39 (6.4%)
	Agree	80 (13.1%)
	Neither agree nor disagree	127 (20.8%)
	Disagree	234 (38.2%)
	Strongly disagree	132 (21.6%)
Would you consider social egg freezing to allow yourself the opportunity for financial stability prior to motherhood?	Strongly agree	41(6.7%)
	Agree	104 (17.0%)
	Neither agree nor disagree	126 (20.6%)
	Disagree	219 (35.8%)
	Strongly agree	122 (19.9%)
Do you feel that you have not been made aware of the option of social egg freezing available to you sooner, and this has since impacted the likelihood of you pursuing this method of fertility preservation further?	Strongly agree	71 (11.6%)
	Agree	107 (17.5%)
	Neither agree nor disagree	185 (30.2%)
	Disagree	178 (29.1%)
	Strongly disagree	71 (11.6%)
Social egg freezing can significantly prolong a woman’s reproductive years.	Yes	388 (63.4%)
	No	38 (6.2%)
	Unsure	186 (30.4%)
Do you know where the service is available in Saudi Arabia?	Yes	231 (37.7%)
	No	36 (5.9%)
	Unsure	345 (56.4%)
At what age of a woman freezing her eggs for social reasons, would result in the highest predicted chance of a successful livebirth later in life when she then attempts to use the eggs to get pregnant?	20-25	152 (24.8%)
	26-30	186 (30.4%)
	31-35	114 (18.6%)
	36-39	40 (6.5%)
	Unsure	120 (19.6%)
One cycle of treatment is always sufficient to retrieve enough eggs for freezing.	True	74 (12.1%)
	False	81 (13.2%)
	Unsure	457 (74.7%)
The process of egg freezing will pose risks to the woman’s health and future fertility.	True	48 (7.8%)
	False	263 (43.0%)
	Unsure	301 (49.2%)
Frozen eggs are guaranteed to result in pregnancy in the future.	True	129 (21.1%)
	False	164 (26.8%)
	Unsure	319 (52.1%)

**Figure 2:**
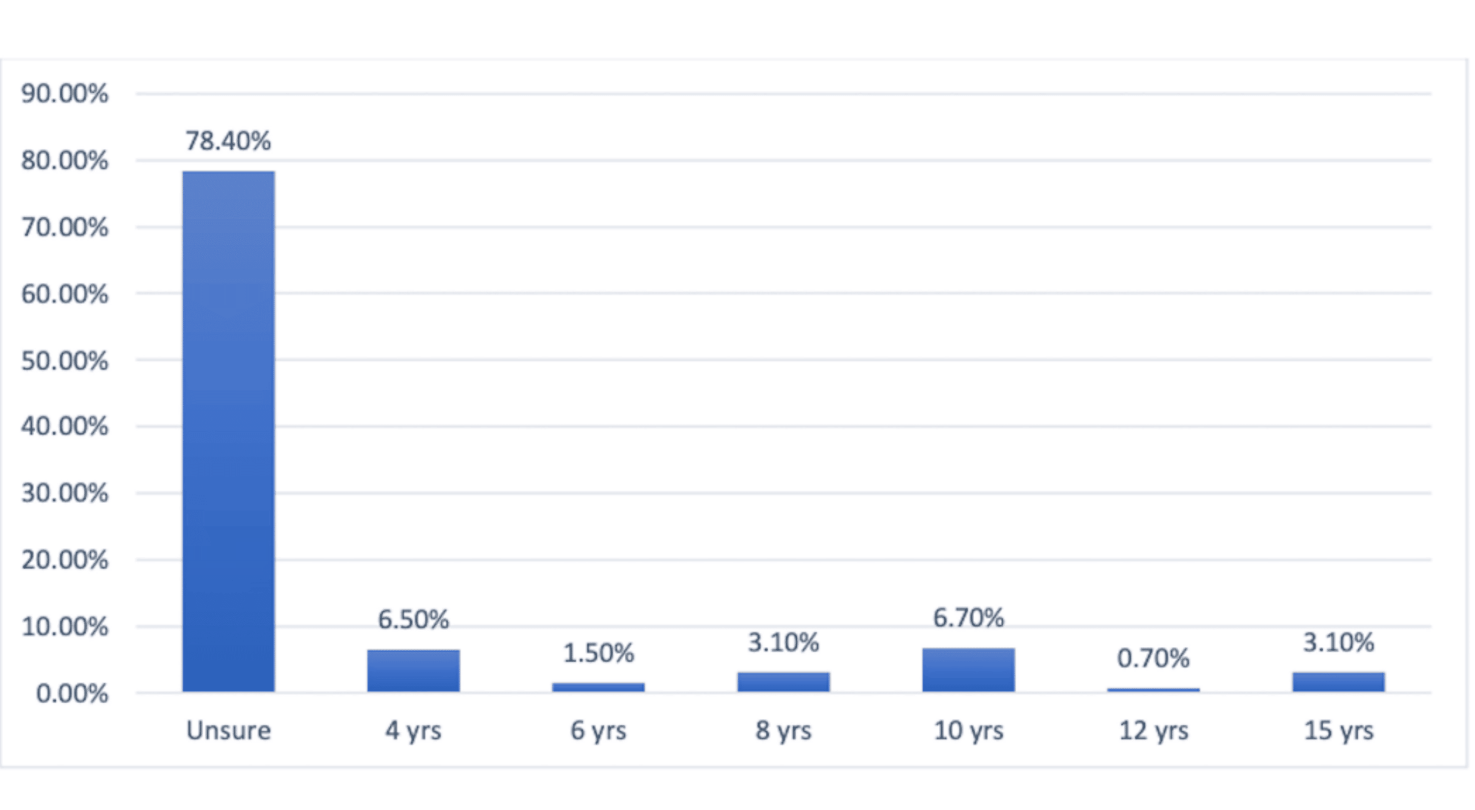
Knowledge of the maximum period eggs can be frozen and stored.

The majority of participants (n=480; 78.4%) were unaware of the legal maximum for freezing and storing eggs. Just a small portion of participants gave precise responses: 40 (6.5%) participants said four years, nine (1.5%) said six years, 19 (19.1%) said eight years, 40 (6.7%) said 10 years, four (0.7%) said 12 years, and 19 (3.1%) said 15 years (Figure 2).

**Figure 3:**
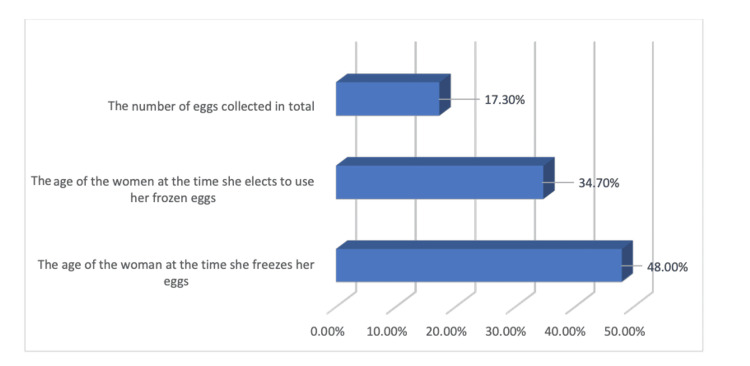
Knowledge of the strongest predictor of a successful pregnancy.

A total of 294 (48.0%) of the respondents thought that a woman's age at the time she freezes her eggs is the best indicator of a healthy pregnancy. Subsequently, 212 (34.7%) thought it was the woman's age when she decided to use her frozen eggs. Finally, 106 people (17.3%) thought the best predictor was the total number of eggs collected (Figure 3).

The multinomial regression analysis results for predictors of KAP regarding OC among 612 participants revealed that age did not significantly predict KAP outcomes. Education level was a significant predictor, with higher education (Master’s and PhD) associated with better knowledge (OR: 2.36, 2.65), more positive attitudes (OR: 2.50, 2.85), and improved practices (OR: 2.20, 2.40) compared to a Bachelor's degree. Diploma holders also showed better knowledge (OR: 0.37) and practices (OR: 0.45) than those with secondary education or below. Employment status and marital status were not significant predictors of KAP outcomes (Table 6).

**Table 6:** Predictors of knowledge, attitude, and practice regarding oocyte cryopreservation The reference category for multinomial regression is indicated for each variable. *Statistically significant at p < 0.05.

Variables	Category	Knowledge, OR (95% CI)	Attitude, OR (95% CI)	Practice, OR (95% CI)	P-value*
Age	18-25 years	1.00 (reference)	1.00 (reference)	1.00 (reference)	0.627
	26-30 years	1.13 (0.55-2.33)	0.98 (0.48-2.00)	1.24 (0.57-2.69)	
	31-35 years	0.95 (0.46-1.96)	1.05 (0.51-2.17)	0.88 (0.41-1.90)	
	36-39 years	1.03 (0.49-2.16)	0.92 (0.43-1.97)	1.17 (0.52-2.65)	
	40-45 years	0.89 (0.43-1.84)	1.15 (0.55-2.38)	0.96 (0.43-2.13)	
	46-50 years	1.16 (0.55-2.43)	1.25 (0.58-2.68)	1.08 (0.48-2.42)	
Education	No formal Education	0.22 (0.04-1.09)	0.30 (0.06-1.45)	0.25 (0.05-1.19)	0.004
	Secondary school and below	0.67 (0.34-1.32)	0.75 (0.38-1.49)	0.63 (0.31-1.30)	
	Diploma	0.37 (0.17-0.81)	0.42 (0.19-0.92)	0.45 (0.21-0.97)	
	Bachelor’s Degree	1.00 (reference)	1.00 (reference)	1.00 (reference)	
	Master’s degree	2.36 (1.11-5.00)	2.50 (1.16-5.38)	2.20 (1.00-4.85)	
	PhD	2.65 (1.02-6.88)	2.85 (1.08-7.51)	2.40 (0.88-6.52)	
Employment	Unemployed	0.78 (0.44-1.38)	0.81 (0.46-1.41)	0.85 (0.47-1.54)	0.496
	Employed	1.00 (reference)	1.00 (reference)	1.00 (reference)	
	Student	1.19 (0.67-2.10)	1.10 (0.62-1.95)	1.22 (0.68-2.19)	
	Retired	0.50 (0.09-2.72)	0.55 (0.10-3.07)	0.45 (0.08-2.49)	
Marital Status	Single	1.00 (reference)	1.00 (reference)	1.00 (reference)	0.569
	Married	1.05 (0.62-1.76)	1.10 (0.65-1.85)	1.12 (0.65-1.94)	
	Divorced	0.82 (0.11-6.13)	0.75 (0.10-5.58)	0.90 (0.12-6.66)	
	Widowed	0.40 (0.05-3.09)	0.35 (0.04-2.85)	0.30 (0.04-2.47)	

## Discussion

This study aimed to gain insight into the KAP of women of reproductive age toward OC in the Qassim region. The results highlight several key insights into the reproductive health awareness and preferences of the population studied. The demographic data from Table 1 indicate that the majority of participants were young adults, with 47.1% falling within the 18-25 age group. This demographic trend is noteworthy as it suggests that knowledge and attitudes towards OC could be primarily shaped by younger individuals who are at the beginning of their reproductive years. Similar to findings by Mahesan et al. [11], younger participants often exhibit more openness to and interest in fertility preservation technologies due to their proximity to peak reproductive age and career planning considerations.

A significant portion of the participants held a bachelor’s degree (75.5%), reflecting a relatively high level of education within the sample. This high educational attainment could influence the level of awareness and understanding of reproductive health options, including oocyte cryopreservation. The educational background of the participants underscores the importance of leveraging educational institutions and platforms to disseminate information about fertility preservation. This is consistent with study by Kasaven et al., which also found that higher educational levels are associated with better knowledge and attitudes towards fertility preservation [8]. The employment status of the participants varied, with a notable portion being students (38.6%) and employed individuals (34.3%). This distribution indicates that a significant number of participants are either preparing for or are already in their professional careers. Meanwhile, a smaller segment was unemployed (25.5%), which could influence their access to and prioritization of reproductive health services. The diversity in employment status highlights the need for flexible and accessible fertility preservation options that can cater to different socioeconomic backgrounds. Similar findings were reported by Akhondi et al. [12], indicating that career stage and employment status can impact reproductive health decisions and access to services. Marital status distribution showed that over half of the participants (56.4%) were single, while 42.0% were married. Very few individuals were widowed (1.3%) or divorced (0.3%). This demographic profile suggests that the majority of participants are likely in the planning stages of their reproductive lives, making them ideal candidates for interventions aimed at improving knowledge and attitudes towards fertility preservation. Understanding the marital status of participants can help tailor educational messages to address specific concerns and aspirations related to family planning and reproductive health.

The study reveals significant reproductive aspirations among participants, with 68.1% expressing a desire to have children (Table 2). This high percentage underscores the importance of understanding and addressing reproductive health needs in the population. The average desired age for conceiving the first child was between 25-29 years, and for the last child, it was between 36-39 years. This planned age range reflects a common trend where individuals aim to balance their career and personal life goals before starting a family. By planning to have their first child in their mid to late 20s, participants are likely considering the completion of their education, establishment of a career, and attainment of financial stability. The desire to have the last child by the late 30s further illustrates an awareness of the biological constraints on fertility and the potential complications associated with advanced maternal age. Additionally, 9.3% of participants reported having medical conditions affecting fertility. This finding highlights the need for heightened awareness and resources for fertility preservation among those with health challenges. Medical conditions such as polycystic ovary syndrome (PCOS), endometriosis, and other reproductive health disorders can significantly impact fertility. Providing information and resources for fertility preservation, such as OC, can help individuals with such conditions plan their reproductive futures more effectively. This finding is consistent with research by Akhondi et al., which highlights the growing trend of delayed childbearing and its implications for fertility [12]. Delayed childbearing, often influenced by career aspirations and personal goals, can lead to a decline in fertility due to the natural decrease in the quantity and quality of oocytes as women age. Akhondi et al. emphasize the need for awareness and proactive measures in fertility preservation to mitigate the risks associated with delayed childbearing [12].

Knowledge about fertility varied significantly among participants. While 35.6% correctly identified that women are most fertile between 20-24 years, a substantial number (14.7%) were unaware of the age at which fertility begins to decline. This lack of awareness is concerning because it indicates that many individuals may not fully understand the optimal timeframe for conception, which can impact their reproductive planning and decisions. Only 0.5% recognized the age range of 36-39 as critical for declining fertility, underscoring a significant gap in awareness. This lack of recognition is problematic as it suggests that the majority of participants are not aware of the more immediate and severe decline in fertility that occurs in the late thirties. Educating the public about these critical periods is essential for helping individuals make informed reproductive choices. Most participants understood that fertility typically declines between 40-45 years, with 53.9% identifying this age range. While this shows a better awareness of the overall decline in fertility with age, it also highlights the need for more specific education about the earlier onset of fertility decline and the biological factors affecting reproductive health. The study found a high level of awareness regarding the risks associated with advanced maternal age, with 79.4% correctly identifying the age range that poses a higher risk. Additionally, 87.6% were aware of health complications that could arise from late pregnancies, such as increased risk of gestational diabetes, hypertension, and chromosomal abnormalities like Down syndrome. This high level of awareness indicates that participants are generally knowledgeable about the risks associated with delaying pregnancy. Despite this awareness, only 17.0% were unwilling to delay pregnancy due to these risks, indicating a potential gap between knowledge and action. This gap suggests that while individuals are aware of the risks, other factors, such as career goals, personal readiness, and societal pressures, may influence their decision to delay pregnancy. This is consistent with findings by Mahesan et al., which showed that high awareness did not always translate into proactive reproductive planning behaviours [11]. They found that although many participants were aware of fertility decline and associated risks, their reproductive behaviors did not always reflect this knowledge, indicating a complex interplay of factors influencing reproductive decisions.

Participants' perceptions towards fertility preservation, particularly social egg freezing, were mixed, indicating varying levels of awareness and concern about fertility issues. While a significant majority (87.6%) acknowledged that medical conditions could significantly impact reproductive years, only 22.1% expressed concern about declining fertility. This discrepancy suggests that many participants may underestimate the impact of age and health on fertility, potentially due to a lack of comprehensive information or a perception that fertility preservation is not immediately relevant to their personal circumstances. The low level of concern about declining fertility highlights a critical gap in awareness. It suggests that many individuals might not fully understand the implications of delayed childbearing or the potential benefits of proactive fertility preservation. This gap could result from societal norms that prioritize career and personal development over early family planning, or it could indicate a need for more effective communication about the biological realities of fertility decline. 52.3% of participants believed that gynaecologists and general practitioners should proactively discuss family planning and fertility preservation options with their patients. This preference for professional guidance underscores the trust placed in healthcare providers and the critical role they play in disseminating fertility-related information. Participants' desire for proactive discussions from healthcare professionals indicates a need for these conversations to be more routine and comprehensive in clinical settings. This finding aligns with the research by Taniskidou et al., which emphasizes the importance of healthcare professionals in educating patients about fertility preservation [13]. They highlighted that patients often rely on their healthcare providers for accurate and personalized information, and proactive communication from these professionals can significantly influence patients' understanding and decisions regarding fertility preservation. The study by Taniskidou et al. also found that when healthcare providers discuss fertility preservation, patients are more likely to consider and utilize these options, reflecting the profound impact of professional guidance on patient decisions [13].

Awareness of social egg freezing among participants was notably high, with 88.1% having heard about the procedure. However, only 28.2% felt knowledgeable about it, indicating a significant information gap. This disparity between awareness and knowledge suggests that while the concept of social egg freezing is well-known, many individuals lack a detailed understanding of the procedure, its benefits, and its limitations. Regarding the perceived benefits of social egg freezing, a majority of participants (63.4%) believed that it could extend reproductive years. This perception aligns with the primary medical rationale behind the procedure, which is to preserve a woman's fertility potential by freezing her eggs at a younger age when they are typically healthier. Additionally, 29.4% of participants strongly agreed that social egg freezing could reduce the pressure to procreate, particularly in supporting career planning and personal development. This reflects a growing recognition of the procedure's potential to offer women more flexibility in balancing their reproductive goals with their professional and personal aspirations. Despite this recognition, the substantial information gap highlights the need for enhanced education and support regarding social egg freezing. Many participants might be aware of the procedure but do not fully understand the specifics, such as the optimal age for egg freezing, the success rates, potential risks, costs, and long-term implications. Bridging this knowledge gap is essential to enable individuals to make informed decisions about their reproductive health. Similar sentiments were echoed in studies by Matevossian et al., which found a gap between awareness and in-depth knowledge among medical trainees [14]. They highlighted that even among individuals with a medical background, there was a lack of comprehensive understanding of fertility preservation techniques. This underscores the broader issue that general awareness does not necessarily equate to informed decision-making. The findings from this study and those by Matevossian et al. [14] suggest that more robust educational initiatives are needed. These should focus on providing detailed, accessible information about social egg freezing, addressing common misconceptions, and highlighting both the benefits and limitations. A study from the United Arab Emirates also reached the conclusion that despite the awareness of women about this procedure there is a need to provide accurate information by physicians [15]. A study from Turkey also reported that women lack awareness about reproductive ageing and the optimal timing for requesting cryopreservation [16]. Therefore, healthcare providers play a crucial role in this educational effort. By integrating detailed discussions about social egg freezing into routine gynaecological consultations and offering resources such as informational pamphlets, webinars, and counselling sessions, healthcare professionals can help bridge the knowledge gap [14].

This study identified a statistically significant relationship between education level and knowledge of OC (P=0.004). Participants with higher education levels, specifically those holding bachelor’s, master’s, and PhD degrees, exhibited significantly greater knowledge about OC compared to those with lower levels of education. This finding underscores the pivotal role that education plays in enhancing awareness and understanding of fertility preservation options. The influence of education on reproductive health knowledge suggests that targeted educational interventions could be highly effective in bridging existing knowledge gaps. Educational campaigns, particularly those integrated into higher education curricula and public health initiatives, can provide crucial information about OC. Such efforts could include seminars, informational brochures, and digital content tailored to various educational levels to ensure broad and effective dissemination of knowledge. The significant role of education in shaping reproductive health knowledge aligns with previous research findings. For instance, Mahesan et al. found that higher education levels were associated with increased awareness and understanding of elective OC among undergraduate and medical students [9]. Similarly, Kasaven et al. highlighted that women with higher educational attainment were more likely to be knowledgeable about fertility decline and the benefits of elective OC [6]. Both studies emphasize that educational attainment is a crucial determinant of fertility awareness and attitudes. These findings suggest that enhancing educational efforts around reproductive health, particularly targeting those with lower educational backgrounds, could improve overall awareness and understanding of OC. By doing so, individuals would be better equipped to make informed decisions about their reproductive futures. This study's results resonate with broader trends in health education, where higher educational attainment often correlates with better health literacy and proactive health behaviours. As such, incorporating comprehensive reproductive health education into both formal education systems and public health initiatives could play a significant role in promoting fertility preservation knowledge and practices.

The study has a limitation of being conducted only in the Al-Qassim region. But we think that being a central region of Saudi Arabia, the situation will be similar in other regions of the country. Another limitation is that an online survey is mostly answered by educated and younger population who has access to internet. 

## Conclusions

The study highlights the influence of educational attainment on knowledge of OC. These findings align with broader trends in health education, suggesting that higher educational attainment correlates with better reproductive health education and proactive health behaviours. There is a need to educate women more about the available fertility preservation options. Enhancing health literacy through educational programs may lead to more proactive health behaviours and better reproductive outcomes.

## Appendices

Questionnaire

There are three sections Section 1) About you Section 2) Current knowledge of fertility Section 3) Perceptions of fertility preservation Section 4) Knowledge of social egg freezing In total, the questionnaire will take you approximately 10-15 minutes to complete. You can pause and return to the questionnaire multiple times. You may skip questions or entire sections if you do not wish to provide a response. Just place a tick √ on your choices

**Table 7:** Section 1a: Socialdemographical information of the participants

Questions	Category
Age	18-25 years
	26-30 years
	31-35 years
	36-39 years
	40-45 years
	46-50 years
Education	No formal Education
	Secondary school and below
	Diploma
	Bachelor’s Degree
	Master’s degree
	PhD
Employment	Unemployed
	Employed
	Student
	Retired
Marital Status	Single
	Married
	Divorced
	Widowed
Place of Residence	Qassim
	Non-Qassim

**Table 8:** Section 1b: Participant’s reproductive aspirations

Questions	Category
Do you have children?	Yes
	No
How many children do you have?	Write the number here
Do you wish to have children in future?	Yes
	No
	Unsure
What is your desired age to have your first child (if not yet had children)?	20-24
	25-29
	30-35
	36-39
	40-45
	>45
	Unsure
	I had my 1^st^ child
What is your desired age to have your last child?	20-24
	25-29
	30-35
	36-39
	40-45
	>45
	Unsure
Have you ever been diagnosed with a health problem that could compromise your fertility?	Yes
	No
	Unsure

**Table 9:** Section 2: Participant’s knowledge of fertility

QUESTIONS	Category
What age are women most fertile?	15-19
	20-24
	25-29
	30-35
	36-39
	40-45
	Unsure
At what age on average do you think a woman’s fertility begins to decline?	15-19
	20-24
	25-29
	30-35
	36-39
	40-45
	Unsure
What are the chances a woman of 30 years old will become pregnant after one year of unprotected sexual intercourse?	<10%
	10-19%
	20-39%
	40-59%
	60-79%
	80-100%
What are the chances a woman of 40 years old will become pregnant after a year of unprotected sexual intercourse?	<10%
	10-19%
	20-39%
	40-59%
	60-79%
	80-100%
From what age do you think doctors consider a pregnant woman ‘high risk’ due to advanced maternal age?	15-19
	20-24
	25-29
	30-35
	36-39
	40-45
	Unsure
Are you aware that getting pregnant at an advanced maternal age can be associated with greater risk of high blood pressure in pregnancy, blood clots in legs and lungs and your baby being born prematurely?	Yes
	No
	Unsure

**Table 10:** Section 3: Perceptions of fertility preservation

QUESTIONS	Category
Can a woman’s number of reproductive years be significantly shortened by an underlying health condition?	Yes
	No
	Unsure
I am currently worried about my declining fertility due to advanced age or a health-related problem:	Strongly agree
	Agree
	Neither agree nor disagree
	Disagree
	Strongly disagree
Having my own biological child is important to me:	Strongly agree
	Agree
	Neither agree nor disagree
	Disagree
	Strongly disagree
I am prepared to become pregnant at a later age, despite the additional risks associated with advanced maternal age during pregnancy:	Strongly agree
	Agree
	Neither agree nor disagree
	Disagree
	Strongly disagree
Gynaecologists/GP’s should initiate discussion regarding family planning with all female patients of reproductive age, even if the patient does not mention it herself:	Strongly agree
	Agree
	Neither agree nor disagree
	Disagree
	Strongly disagree
I currently feel well-informed regarding the options available to me to preserve my fertility in case of age or health-related fertility decline:	Strongly agree
	Agree
	Neither agree nor disagree
	Disagree
	Strongly disagree

**Table 11:** Section 4: Knowledge of elective oocyte cryopreservation

QUESTIONS	Category
Have you heard of social egg freezing?	Yes
	No
I would consider freezing my eggs to prevent age-related fertility decline?	Strongly agree
	Agree
	Neither agree nor disagree
	Disagree
	Strongly disagree
If social egg freezing was available to me, it would reduce the pressure for me to have children?	Strongly agree
	Agree
	Neither agree nor disagree
	Disagree
	Strongly disagree
Should social egg freezing be made accessible to all by the Ministry of Health?	Yes
	No
	Unsure
Would you consider social egg freezing if you want to delay having a child?	Strongly agree
	Agree
	Neither agree nor disagree
	Disagree
	Strongly disagree
Would you consider social egg freezing to focus on your career?	Strongly agree
	Agree
	Neither agree nor disagree
	Disagree
	Strongly disagree
Would you consider social egg freezing to allow yourself the opportunity for financial stability prior to motherhood?	Strongly agree
	Agree
	Neither agree nor disagree
	Disagree
	Strongly agree
Do you feel that you have not been made aware of the option of social egg freezing available to you sooner, and this has since impacted the likelihood of you pursuing this method of fertility preservation further?	Strongly agree
	Agree
	Neither agree nor disagree
	Disagree
	Strongly disagree
Social egg freezing can significantly prolong a woman’s reproductive years?	Yes
	No
	Unsure
Do you where the service is available in Saudi Arabia?	Yes
	No
	Unsure
At what age of a woman freezing her eggs for social reasons, would result in the highest predicted chance of a successful livebirth later in life when she then attempts to use the eggs to get pregnant?	20-25
	26-30
	31-35
	36-39
	Unsure
One cycle of treatment is always sufficient to retrieve enough eggs for freezing?	True
	False
	Unsure
The process of egg freezing will pose risks to the woman’s health and future fertility?	True
	False
	Unsure
Frozen eggs are guaranteed to result in pregnancy in the future?	True
	False
	Unsure

Please leave any comments that you have for the research team: Thank you for taking part.
